# Unintentional Genomic Changes Endow *Cupriavidus metallidurans* with an Augmented Heavy-Metal Resistance

**DOI:** 10.3390/genes9110551

**Published:** 2018-11-13

**Authors:** Felipe A. Millacura, Paul J. Janssen, Pieter Monsieurs, Ann Janssen, Ann Provoost, Rob Van Houdt, Luis A. Rojas

**Affiliations:** 1 School of Biological Sciences, University of Edinburgh, Edinburgh EH9 3JQ, UK; s1647595@sms.ed.ac.uk; 2 Interdisciplinary Biosciences, Belgian Nuclear Research Centre, SCK•CEN, 2400 Mol, Belgium; pjanssen@sckcen.be (P.J.J.); pieter.monsieurs@sckcen.be (P.M.); ann.janssen@sckcen.be (A.J); ann.provoost@sckcen.be (A.P.); rvhoudto@sckcen.be (R.V.H.); 3 Chemistry Department, Faculty of Sciences, Universidad Católica del Norte, UCN, Antofagasta 1240000, Chile

**Keywords:** *Cupriavidus*, heavy metals, genomic islands, genomic rearrangements, metal resistance genes

## Abstract

For the past three decades, *Cupriavidus metallidurans* has been one of the major model organisms for bacterial tolerance to heavy metals. Its type strain CH34 contains at least 24 gene clusters distributed over four replicons, allowing for intricate and multilayered metal responses. To gain organic mercury resistance in CH34, broad-spectrum *mer* genes were introduced in a previous work via conjugation of the IncP-1β plasmid pTP6. However, we recently noted that this CH34-derived strain, MSR33, unexpectedly showed an increased resistance to other metals (i.e., Co^2+^, Ni^2+^, and Cd^2+^). To thoroughly investigate this phenomenon, we resequenced the entire genome of MSR33 and compared its DNA sequence and basal gene expression profile to those of its parental strain CH34. Genome comparison identified 11 insertions or deletions (INDELs) and nine single nucleotide polymorphisms (SNPs), whereas transcriptomic analysis displayed 107 differentially expressed genes. Sequence data implicated the transposition of IS*1088* in higher Co^2+^ and Ni^2+^ resistances and altered gene expression, although the precise mechanisms of the augmented Cd^2+^ resistance in MSR33 remains elusive. Our work indicates that conjugation procedures involving large complex genomes and extensive mobilomes may pose a considerable risk toward the introduction of unwanted, undocumented genetic changes. Special efforts are needed for the applied use and further development of small nonconjugative broad-host plasmid vectors, ideally involving CRISPR-related and advanced biosynthetic technologies.

## 1. Introduction

Since life appeared on Earth some 3.7 billion years ago, microorganisms have undergone molecular changes to adapt (i.e., respond to selection) to the harsh conditions of their natural habitats, including extreme temperatures, pH, salinity, UV and ionizing radiation, and heavy metals [1]. In addition, global fluctuations in the composition of the atmosphere, the oceans, and the earth crust have elicited genomic changes in microbes over eons of time and hence contributed to microbial diversity [2]. Certain microorganisms had been adapted to heavy metals and radionuclides prior to human appearance. However, during the past few hundred years, anthropogenic influences have forced microbes to also adapt to pollutants previously nonexistent (xenobiotics) [3,4,5,6], as well as to increased concentrations of heavy metals and radionuclides [7,8,9].

Members of the beta-proteobacterial genus *Cupriavidus* are prime examples of microbial endurance, possessing a variety of genomic islands involved in the resistance to heavy metals or the degradation of aromatics or xenobiotics [10,11,12,13,14,15,16,17,18,19,20]. They all typically display a bipartite chromosomal structure with one chromosomal replicon bearing the marks of a plasmid-type maintenance and replication system henceforth called “chromid”. In addition, most *Cupriavidus* strains carry one or two dispensable megaplasmids with a size of 100 kb or more. The model organism for heavy metal resistance, *Cupriavidus metallidurans* strain CH34, carries two megaplasmids, pMOL28 and pMOL30. Together, the four CH34 replicons encode resistance markers for a plethora of heavy metals including copper, nickel, zinc, cobalt, cadmium, chrome, lead, silver, gold, mercury, caesium, selenium, strontium, and uranium [12,21,22,23,24,25]. These resistances are mainly related to a variety of metal reduction and efflux systems [26,27,28]. Aromatics degradation, on the other hand, is carried out by various bacterial multicomponent mono- and di-oxygenases solely encoded by genes on the chromosome and chromid [29].

In an effort to improve the inorganic and organic mercury resistance of *C. metallidurans* CH34 and thus improve its utility in cleaning up mercury-contaminated environments, the IncP-1β plasmid pTP6, providing additional *mer* genes [30], was introduced in CH34 by biparental mating, leading to strain MSR33 [31]. These extra *mer* genes are part of a transposon, Tn*50580*, which is necessary for broad-spectrum (organomercury) resistance, including two genes, *merG* and *merB*, not native to CH34 (i.e., CH34 only contains two narrow-spectrum mercury resistance *merRTPADE* operons, one on each megaplasmid, conferring resistance to inorganic mercury). MerB plays a key role in methylmercury degradation through its unique ability to cleave the carbon-mercury bond in methylmercury and the subsequent shuttling of ionic mercury to MerA to reduce it to the less harmful elemental mercury.

In comparison to its parental strain CH34, strain MSR33 became 240% more resistant to inorganic mercury and gained resistance to methylmercury by incorporating the previously nonpresent *merBG* genes. Other metal resistances (as tested for chrome and copper) were seemingly unaffected, and the pTP6 plasmid was stably maintained for over 70 generations under nonselective pressure [31]. However, when we recently tested the resistances for both strains to additional metals (i.e., cadmium, cobalt, and nickel), we noted a significant increase of metal resistance for strain MSR33 compared to CH34 (this study). As this was fully unexpected, since the only difference between the two strains should be an additional *mer* gene dosage, implicating only an improved mercury resistance, we set out to investigate the reasons for this phenomenon. Considering the genome plasticity [32,33,34] and the intricate relationships between metal resistance loci [24] in *C. metallidurans* CH34, we decided to sequence the full genome of strain MSR33 and compare the sequence of its replicons with the corresponding replicons of the parental strain CH34 and with plasmid pTP6. We also performed microarray-based expression analysis on both CH34 and MSR33 gene sets to determine whether genomic differences could be correlated with differences in the expression of individual genes.

## 2. Materials and Methods

### 2.1. Strains and Culture Conditions

*Cupriavidus metallidurans* strains CH34 and MSR33, obtained from respectively the SCK•CEN (Mol, Belgium) and the Univesidad Católica del Norte (UCN) (Antofagasta, Chile) culture collections, were cultivated at 30 °C and 200 revolutions per minute (rpm) on a shaker in dark, aerobic conditions in a Tris-buffered mineral medium (MM284) [35] with 0.4% (*w*/*v*) succinate as the sole carbon source. *Escherichia coli* JM109 pTP6 (also obtained from the UCN culture collection) was cultured at 37 °C on an M9 minimal medium [36] supplemented with 0.4% (*w*/*v*) glucose.

### 2.2. Synthetic Construct Generation

The *merTPAGB*_1_ gene cluster of pTP6 was amplified by PCR using Phusion High-Fidelity DNA polymerase (ThermoFisher, Aalst, Belgium) with primer pair pTP6mer_Fw-Rv (Table S1). In tandem, the broad-host-range cloning vector pBBR1MCS-2 [37] was linearized by PCR with the same DNA polymerase and primer pair pBBR1MCS-2_GA_Fw-Rv (Table S1), providing homologous ends with the amplified *merTPAGB*_1_ locus. These compatible PCR products were end-ligated using the Invitrogen GeneArt^®^ Seamless Cloning and Assembly Enzyme Mix (ThermoFisher). After transforming *E. coli* DG1 with the ligation mix and selection on Lysogeny Broth (LB) with 50 μg/mL kanamycin (Km), four randomly chosen transformants were tested by DNA digestion and fragment sizing for correct plasmid construction holding the *merTBAGB*_1_ genes. The plasmid gene construct of one transformant was further verified by sequencing its insert using forward and reverse cloning primers (Table S1) prior to electroporation into *C. metallidurans* CH34 on an Eppendorf 2510 Electroporator (Eppendorf, Aarschot, Belgium) using conditions as described previously [38].

### 2.3. Estimation of Bacterial Tolerance to Metals

Strains CH34 and MSR33 were grown overnight in MM284 liquid media with 0.4% (*w*/*v*) succinate as the sole carbon source and thereafter used as a pre-inoculum (1% *v*/*v*) for a freshly prepared 200 μL culture supplemented with increased concentrations of Hg^2+^ (from 0.0625 mM to 8 mM), Cd^2+^ (from 0.25 mM to 8 mM), and increasing steps (1 mM) of Co^2+^ or Ni^2+^ (from 5 mM to 20 mM). Cultures for metal contact were grown in microtiter plates at 30 °C on a rotary shaker at 120 rpm. Metal ion solutions were prepared from soluble salts of analytical grade (CdCl_2_, HgCl_2_, CoCl_2_·6H_2_O, and NiCl_2_·6H_2_O) in double-deionized water and were filter-sterilized before use. The lowest metal concentration that prevented growth after 48 h (i.e., showing no growth as measured at OD_600_ by a CLARIOstar microplate reader (BMG LabTech, Offenburg, Germany)), was considered the minimum inhibitory concentration (MIC) (Table 1 and Table S2). All MIC analyses were performed using biological triplicates.

### 2.4. Plasmid Copy Number Determination

Single-copy (i.e., “replicon-unique”) genes were taken as representatives of the chromosome (*cadA*), chromid (*zniA*), and plasmids pMOL30 (*nccA*), pMOL28 (*cnrA*), and pTP6 (*merG*). Primer pairs were designed to amplify 150 bp of each gene (Table S1). Real-time PCR was performed on a 7500 Applied Biosystems Fast Real-Time PCR System (ThermoFisher) using QiaGen RT^2^ Sybr Green Rox qPCR Mastermix (ThermoFisher), 20 ng of MSR33 or CH34 genomic DNA as a template, and 0.2 μM of each primer. To reduce nonspecific amplification, we incubated this mixture at 95 °C for 10 min as part of a hot start PCR setup. Next, a 40-cycle amplification and quantification protocol (15 s at 95 °C, 15 s at 58 °C, and 30 s at 60 °C) was performed with a single fluorescence measurement for each cycle. Finally, a melting curve program (15 s at 95 °C, 60 s at 60 °C, 30 s at 95 °C, and 15 s at 60 °C) was carried out. Plasmid copy numbers for both strains were determined using the absolute method (allowing estimates of both the absolute and relative number of plasmids per cell), following earlier described protocols [39]. Standard curves were created with 7500 Fast Software v2.3 of Applied Biosystems (Foster City, CA, USA), using serial (10-fold) dilutions of genomic DNA in a linear range from 20 ng to 0.2 pg. The qPCR efficiencies were calculated from slopes of the log-linear portion of the calibration curves from the equation *E* = 10^(1/*slope*)^. Using the linear equation obtained from each calibration curve, log DNA copy numbers were derived by intersecting the obtained *C_t_* values. All analyses were done in triplicate.

### 2.5. Illumina Sequencing and Assembly

MSR33 cells were grown overnight in MM284 liquid medium, and genomic DNA was extracted by using the Qiagen QIAamp DNA Mini Kit (ThermoFisher), following the instructions of the manufacturer. DNA quantity and quality were measured using a DropSense (Trinean, Piscataway, NJ, USA). Genome sequencing was performed on a HiSeq 2500 apparatus (Illumina, San Diego, CA, USA) using 2 × 250 bp paired-end reads. The reads were trimmed using the Trimmomatic tool [40] and their quality assessed using in-house Perl and shell scripts in combination with SAMtools [41], BEDTools [42], and a Burrows–Wheeler aligner with maximum exact matches (bwa-mem) [43].

The entire genome of *C. metallidurans* CH34 was sequenced and largely annotated [12,22]. Sequences for a chromosome (NC_007973.1), a chromid (NC_007974.2), and the large plasmids pMOL28 (NC_007972.2) and pMOL30 (NC_007971.2) were all obtained from GenBank and used as a reference for the assembly and annotation of trimmed sequences of the MSR33 genome. The DNA sequence of pTP6 plasmid, also obtained from Genbank (AM048832), was used for sequence comparisons between the CH34 and MSR33 sequence data sets. The full *C. metallidurans* MSR33 genome sequence (this study) is available from the NCBI Sequence Read Archive (SRA) under accession number PRJNA493617.

### 2.6. Total RNA Isolation and Microarray

*Cupriavidus metallidurans* MSR33 and CH34 cells were both cultivated in triplicate on MM284 medium supplemented with 0.4% (*w*/*v*) succinate. Cell samples were taken at the middle exponential phase (OD_600_ 0.6–0.7) and centrifuged at 16,000 × *g* and 4 °C for 5 min. Pellets were quick-frozen with liquid nitrogen and kept at −80 °C for further analysis. Total RNA extraction was performed as described previously [24] by using an SV Total RNA Isolation System kit (Promega Benelux, Leiden, the Netherlands) according to the manufacturer’s recommendations. Samples were cleaned and concentrated using a Nucleospin RNA cleanup XS kit (Macherey-Nagel, Düren, Germany). Concentrated RNA samples (10–20 μg) were retrotranscribed using the Invitrogen Superscript^TM^ Direct cDNA Labeling System (ThermoFisher) and labeled by incorporation of Cy3-dCTP (ref PA53021, control condition) and Cy-5dCTP (ref PA55021, experimental condition) by Pronto!^TM^ Long Oligo/cDNA Hybridization Solution (supplied with the Corning^®^ Pronto! Universal Microarray Hybridization kit from Merck/Sigma-Aldrich, Overijse, Belgium), following the manufacturer’s instructions. The microarrays we used were designed with 60-*mer* probes for 6205 Open Reading Frames that were spotted in triplicate onto glass slides (UltraGPS, Corning, NY, USA) using a MicroGrid system (BioRobotics, Cambridge, UK) at the microarray platform at SCK•CEN (Mol, Belgium). The spotted slides were cross-linked and placed in the presoaking solutions from the Pronto Kit (Promega, Madison, WI, USA). Analyses were performed on RNAs retrieved from CH34 and MSR33 cells, using respectively Cy3-dCTP and Cy5-dCTP incorporation and determination of Cy3/Cy5 signal intensity ratios. Labeled cDNA was resuspended in the universal hybridization buffer (Pronto kit), mixed, and added to the spotted slide for overnight hybridization at 42 °C in a Tecan HS4800 Pro hybridization station (Tecan Group Ltd., Männedorf, Switzerland). Afterwards, the slide was washed according to Pronto kit’s protocol. Slides were scanned (at 532 and 635 nm) using the GenePix Personal 4100A microarray scanner (Molecular Devices, San Jose, CA, USA). All post-hybridization analyses were performed as described before [24]. In brief, spot signals were qualified using GenePix Pro v.6.0.1 software, and raw median density data were imported into R version 3.3.2 (https://cran.rstudio.com/) for statistical analysis using the LIMMA package version 2.15.15 (http://bioinf.wehi.edu.au/limma/), as available from Bioconductor (https://bioconductor.org). Background correction, normalizations, *t*-statistics, and *p*-value corrections were done as before [24]. Only log-transformed expression results with a *p*-value > 0.05 were considered for data interpretation (Table S2).

## 3. Results and Discussion

### 3.1. The Influence of Plasmid pTP6 on Increased Heavy Metal Resistance in MSR33

The *C. metallidurans* strains CH34 and MSR33 have the same genetic background, but MSR33 has, compared to its parental strain CH34, an extra plasmid 54 kb in size [31]. This plasmid, pTP6, is a broad-host-range IncP-1β plasmid originally isolated from mercury-polluted sediments [30]. It carries a transposon with *mer* genes that are not native to CH34 (i.e., *merG*) that encode an organomercurial transporter, and a pair of duplicate *merB* genes that encode periplasmic organomercurial lyases (Table S3). These additional *mer* genes in strain MSR33, via plasmid pTP6, grant this strain a 2.4-fold increased resistance for Hg^2+^ and a 16-fold increased resistance for CH_3_Hg^+^ [31]. In our hands, we noted a much-improved Hg^2+^ resistance for strain MSR33, with a 10-fold increase in comparison to its parental strain CH34 (Table 1). As an added note, in contrast to Rojas et al. [31], who performed MIC analyses on solid media, we performed our MIC analyses in a liquid medium, increasing metal bioavailability. Hence, sensitivity (i.e., the MIC (Hg) for CH34) was 0.05 mM in Rojas et al.’s study [31] but 0.01 mM in our study]. Surprisingly, when we tested MICs for the metals cadmium, nickel, and cobalt, we found a 2-fold increased resistance in MSR33 to Cd^2+^ and Co^2+^ and a 1.2-fold increased resistance to Ni^2+^ (Table 1).

The only genes on pTP6 with relevance to metal resistance are mercury resistance genes situated on transposon Tn*50580* as the two clusters *merR*_1_*TPAGB*_1_ and *merR*_2_*B*_2_*D*_2_*E* [30]. All other genes are typical for the backbones of self-transmissible and promiscuous IncP-1 plasmids from subgroups α and β and are involved in replication (*trfA*, *ssb*), plasmid maintenance, partitioning, control (*kfrABC*, *incC*, *korABC*, *kluAB*, *klcAB*, *kleABEF*, and a remnant of the resolvase gene *parA*), conjugal transfer (*traCDEFGHIJKL*), mating pair formation (*trbABCDEFGHIJKLMNO*), and transposition (*tniABQR* as part of Tn*50580*). Two more genes, *upf30.5* and *upf31.0*, are located downstream of *trbP* and encode, respectively, a putative outer membrane protein and a site-specific methylase (Figure S1). Except for the *upf* genes and the plasmid maintenance, partitioning, and control genes, pTP6 genes have at least one counterpart on one of the replicons of CH34. Taken together, we did not expect the pTP6 genes, other than the above-mentioned *mer* genes, to play any significant role in the augmented metal resistance of strain MSR33. Nonetheless, we decided to generate a new synthetic construct by cloning the *merTPAGB*_1_ gene cluster in the low copy number broad-host-range cloning vector pBBR1MCS-2 [37]. This small plasmid only contained two genes, *rep* and *mob*, involved in, respectively, plasmid replication and mobilization, as well as a kanamycin resistance marker (Km^R^). Strain CH34 transformed with this new construct reached the same level of inorganic mercury resistance as the MSR33 strain but did not show an increase in cadmium resistance (Table 1). From this we deduced that the *mer* genes of pTP6 exerted a positive effect on host resistance to mercury. However, neither the concomitant increase of cadmium resistance in MSR33 nor its higher resistance to nickel and cobalt could be readily explained by the presence of the auxiliary, pTP6-associated mercury resistance genes in this strain.

We also determined the effect of various mixtures containing both mercury and cadmium on the growth of strains CH34 and MSR33. In general, the combination of the two metals was expected to be more toxic to cells than the corresponding metals alone. Indeed, strain CH34 was capable of growing in up to 1 mM of Cd^2+^ when combined with 6.25 μM of Hg^2+^, and cellular growth was diminished beyond these threshold metal concentrations (Table S4). Instead, strain MSR33 was capable of growing in up to 4 mM of Cd^2+^ when combined with 6.25 μM of Hg^2+^ (Table S4). However, even in combination with mercury, cadmium resistance in MSR33 was still twice as high as the cadmium resistance in CH34. Moreover, strain MSR33 showed much higher tolerance to mercury in combination with cadmium, and growth was only affected at the threshold metal concentrations of 125 μM Cd^2+^ and 100 μM Hg^2+^ (Table S4).

To exclude the possibility that the increased resistance in strain MSR33 to Hg^2+^ and Cd^2+^, either alone or in combination, was a mere effect of gene dosage, we also determined the plasmid copy number for all replicons in strain MSR33 and strain CH34 using quantitative PCR (Table S5). The presence of the pTP6 plasmid in strain MSR33 (plasmid copy number (*PCN*) = 1.8) did not alter the relative copy numbers for the chromid and plasmids pMOL28 and pMOL30 with respect to the calculated chromosomal copy number taken as a reference. In addition, the Tn*50580* transposon carrying the broad spectrum *mer* gene cluster on plasmid pTP6 did not transpose (verified by genome sequence analysis present in Section 3.2).

The CH34 strain carries on its genome a total of four *mer* gene clusters: *merRTPA* on the chromosome, one complete *merRTPADE* on both plasmids pMOL28 and pMOL30, and one truncated *merRT∆P*, also on pMOL30 [12]. One could argue that the presence of a single copy of pTP6 in strain MSR33 raises the number of *mer* genes in strain MSR33, with one unit for genes *T*, *P*, *A*, *D,* and *E*, and two units for gene *R* (a second *merR* gene is located on the right-hand part of Tn*50580* on pTP6 [30] (Table S6), but the *merG* or *merB* genes of pTP6 were not considered here as they only play a role in organomercurial resistance). Considering the calculated PCN values of all replicons, the theoretical abundance of these genes increased by roughly 50–78% (Table S6). *MerR* and *merD* are transcriptional regulators that compete for the same operator sequence in the *merR–merT* intergenic region. The *merT* product is an inner membrane protein involved in the transport of Hg^2+^ ions into the cell cytoplasm. The *merP* product is a small periplasmic Hg^2+^-sequestering protein that shuttles Hg^2+^ to the mercurial reductase MerA, which converts it into the significantly less toxic Hg(0) that then is allowed to leave the cell by passive diffusion. The *merE* product, finally, is another inner membrane protein and may play a role in the uptake of both CH_3_-Hg^+^ and Hg^2+^. All these genes appeared to be intact, and there was no reason for us to assume that any of the multiple-copy genes would be dysfunctional or, with respect to each other (from gene to gene or copy to copy), would be differently transcribed or expressed (owing to limitations of gene-specific primer or probe design for multiple copies of these genes in qPCR or hybridization-based microarray procedures, no *mer* gene-specific expression data are available). A plausible explanation for the positive effect of the pTP6 *mer* genes on host mercury resistance could thus lay in the stoichiometry of *mer* gene products, particularly those involved in Hg^2+^ sequestration (*merP*) and transport (*merT/merE*). Nevertheless, the increased resistance to Cd^2+^ in MSR33 cannot be readily explained by a stoichiometric change in *mer* gene products. Also, when a single *merTPAGB* was introduced on a small plasmid into strain CH34, the increased Hg^2+^ resistance was still there, but the increased Cd^2+^ resistance was no longer seen (Table 1). From this we had to conclude that the MSR33 genetic background, besides the extra plasmid pTP6, may actually have differed from the genetic background of its parental strain CH34. In other words, the MSR33 genome had undergone genetic changes leading to an improved resistance to cadmium and possibly also to other metals. Such a genomic adaptation appears to be common to IncP-1 plasmid backbones [44]. In order to get to the bottom of this we decided to determine the DNA sequence of the entire genome of strain MSR33 and register in detail which genetic changes occurred with respect to the reference genomes of strain CH34 and plasmid pTP6.

### 3.2. The C. metallidurans MSR33 Genome Showed Multiple Insertions or Deletions and Single Nucleotide Polymorphisms

**Figure 1:**
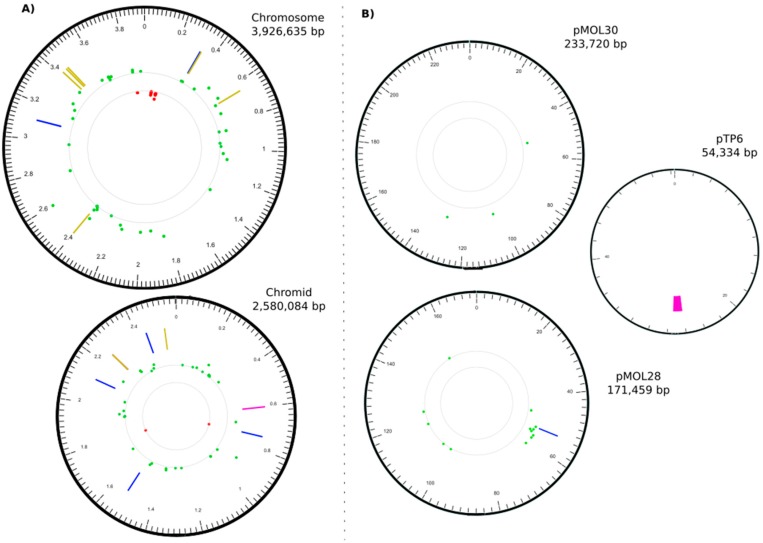
Correlation map between Insertions or Deletions (INDELs), Single nucleotide polymorphisms (SNPs), and transcriptional changes found for *C. metallidurans* MSR33. Concentric circles (ring) displayed from the outside inwards: (**A**) In ring 1, chromosome and chromid size scale in megabases (Mb) using a 20 kilobase (kb) window; (**B**) plasmids size scale in kb using a 2 kb window. In ring 2, position of insertion (blue), deletion (pink), and SNPs (mustard). INDELs and SNPs listed in Table 2 and Table 3. In ring 3, each dot represents a single gene basal expression, overexpression (log_2_ *ratio* > 1, green), or repression (log_2_ *ratio* < −1, red), with a *p*-value < 0.05. Plotted genes listed by function in Table S7. Circos plot created with Circa (http://omgenomics.com/circa).

The whole-genome resequencing of MSR33 (since the known genome sequences of strain CH34 and plasmid pTP6 served as references, we considered this effort as a resequencing project) revealed a total of eight insertions and three deletions (Table 2), and nine single nucleotide polymorphisms (Table 3), all changes being located predominantly across the four replicons of the CH34 backbone, with only one genomic change occurring in plasmid pTP6 (Table 2, Figure 1). Most of the insertions (six out of eight) were found to be related to IS*1088*, an insertion element belonging to the IS*30* family with a typical size range of 1000–1250 bp [45]. It should be noted at this point that the CH34 genome indigenously harboured nine copies of IS*1088*, distributed on its chromosome and chromid but not on its megaplasmids [12], bringing the total of IS*1088* copies in the MSR33 genome to 15.

The majority of the new IS*1088* copies in the MSR33 genome were located on the chromosome and chromid, where all IS*1088* copies indigenous to CH34 resided, but one IS*1088* copy transposed into the *cnrY* gene (Rmet_6205) of pMOL28 (Table 2). This gene was part of the *cnrYXHCBAT* locus involved in the inducible cobalt and nickel resistance in strain CH34, and encoded the anti-sigma factor CnrY that tethered, in conjunction with the sensor protein CnrX, the sigma factor CnrH, but released it in the presence of Ni^2+^ or Co^2+^ [46,47]. The sigma factor CnrH promoted transcription of its own locus *cnrYXH*, but also of the structural locus *cnrCBA*, encoding a resistance nodulation division (RND)-driven efflux system [12,48]. The inactivation of the *cnrY* gene in MSR33 by IS*1088* inevitably led to the constitutive derepression of *cnrCBAT* transcription and explained the increased cobalt and nickel resistance we observed for MSR33 (Table 1) (see also gene expression results in Section 3.3). A similar phenomenon was previously seen for spontaneous mutants of a pMOL30-less CH34 derivative, strain AE126 [35], which showed a significantly increased resistance to cobalt and nickel [49] and which was later acknowledged as being an IS- and frameshift-mediated inactivation of *cnrY* and *cnrX* [50].

The other five genes affected by the insertion of an IS*1088* element were Rmet_0312 (*nptA*) and Rmet_2860 (*tauB*), lying on the chromosome; and Rmet_4160 (*pelF*), Rmet_4867 (*acrA*), and Rmet_5682 (*nimB*), lying on the chromid (Table 2). The first three genes encode proteins with general cellular functions, and their inactivation is very unlikely to affect heavy metal resistance in strain MSR33. The fourth gene, *acrA*, encodes a membrane fusion protein and is part of an intact *acrABC* operon whose gene products form a tripartite multidrug efflux system. The last gene, *nimB*, is involved in efflux-mediated heavy metal resistance, encoding also a membrane fusion protein resembling other membrane metal-binding fusion proteins in structure and function (e.g., CzcB, CnrB, CusB, and ZneB) by forming a periplasmic bridge between the cytoplasmic porter and the outer membrane channel [48]. Nonetheless, taking also into account that the *nimA* gene is already inactivated in strain CH34 (and MSR33) by the presence of the insertion sequence element IS*Rme3* [12], it is hard to see how the inactivation of *nimB* would result in the increased metal resistance we observed in strain MSR33. Two insertions were not attributed to IS*1088*. One appeared to be the result of a Tn*3*-related transposition event affecting gene Rmet_5388, encoding a tentative ApbE-like lipoprotein, while the other concerned an unknown mutational event in gene Rmet_5508 resulting in the insertion of a nucleotide triplet (+CTT) (Table 2). This gene encodes a long-chain fatty-acid CoA-ligase and also underwent a triplet deletion (-CGG) just a few nucleotides downstream of the triplet insert. As a combined result, the actual change at the protein level remained perfectly in-frame and gave a protein of the same length, but led to an altered peptide sequence at positions 149–153 (i.e., Xxx-Leu-**Arg**-**Phe**-**Ala**-Gln-Xxx in CH34 to Xxx-Leu-**Phe**-**Ala**-**Lys**-Gln-Xxx in MSR33 (amino acidic sequence change from **Arg**-**Phe**-**Ala** to **Phe**-**Ala**-**Lys**). The third deletion in MSR33 occurred in plasmid pTP6, effectively destroying the genes *upf30.5*, *upf31.0*, and *parA*, immediately preceding Tn*50580*. Except for *cnrY* and *nimB*, none of the aforementioned genes are in any way associated with metal resistance.

In addition to the eight insertions and three deletions in the MSR33 genome, sequence analysis revealed the presence of nine single nucleotide polymorphisms (Table 3). Two of those occurred in intergenic regions on the chromosome, without apparent disruption of gene regulatory elements, while the other seven occurred in protein-encoding genes (four on the chromosome and three on the chromid). Except for the “silent” mutation (i.e., no aa change) at position 645608, these Single nucleotide polymorphisms (SNPs) caused aa substitutions in the corresponding gene products (Table 3). No SNPs were detected in any of the plasmids. Remarkably, gene Rmet_5508 lying on the chromid (CHR2) once again was a target for mutation, displaying two SNPs in the immediate vicinity of the aforementioned triplet insertion and deletion in this gene (Table 3), bringing the full change of this region from **RFA**QK**P**AY**V** to **FAK**QK**T**AY**E** (changes are in bold and underlined). It is uncertain whether these protein changes would have any effect on the cellular and metabolic functions in MSR33.

**Figure 2:**
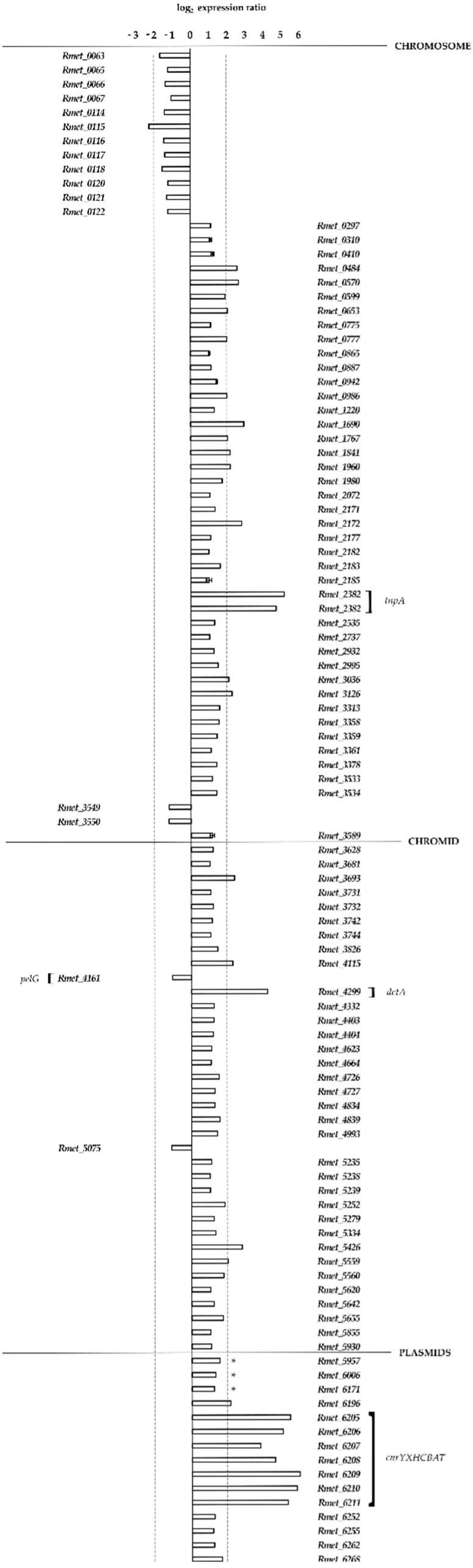
Transcriptional changes in *C. metallidurans* MSR33 with respect to CH34, with both strains grown under equal and nonselective conditions (see methods). Bar graphs show the significantly (*p*-value < 0.05) higher expression (log_2_ ratio > +1) and lower expression (log_2_ ratio < −1) of MSR33 genes (with CH34 gene expression levels as reference). Transcriptional changes from genes pertaining to all replicons are depicted. Genes indicated with an * are part of pMOL30.

Taken together, of the 11 Insertions or Deletions (INDELs) and nine SNPs identified by the whole genome resequencing of the *C. metallidurans* strain MSR33, only the *cnrY* inactivation by IS*1088* and the concomitant derepression of *cnrB* (see above) may be directly linked to the observed augmented heavy metal resistance in this strain, at least for Co^2+^ and Ni^2+^ (see above). None of the other genomic changes seemed to play a role in this augmentation. The augmented resistance for Cd^2+^ in strain MSR33 (Table 1), however, remains a puzzle. The fact that such augmentation for Cd^2+^ was only noted for MSR33 with an altered genome (i.e., with 17 INDELs and six SNPs), but not in CH34 transformed with plasmid pBBR::*merTPAGB*_1_ (Table 1) strongly indicates that the MSR33 genetic background was at play. In basic terms, bacterial resistance to toxic metals depends on two cellular processes, metal binding and metal transport, with the former generally being an intrinsic part of the latter. It is well established that many proteins or peptides that mediate the transport, buffering, or detoxification of metal ions in living cells have metal-binding domains (MBDs) in which certain amino acid residues (e.g., cysteine), as well as their structural layout and relative position to each other, play a key role in metal selectivity and specificity [51,52]. While some of these proteins might be highly metal-specific, other proteins follow a more relaxed, nonspecific mode of metal binding. For instance, divalent metal uptake in *C. metallidurans* is governed by a battery of redundant transporters that display a minimal degree of metal cation selectivity [53]. Depending on the environment, this may lead to a cytoplasmic pool of unsolicited metal ions that at some point, particularly when reaching a toxic threshold, need to be removed by the cell. In *C. metallidurans* this was done by one of three efflux systems: Cation diffusion facilitators (CDF), P-type ATPases, and the earlier mentioned RND-driven transenvelope transporters (HME-RND). Their main task in *C. metallidurans*, because of this bacterium’s adaptation to metal-rich environments, was to balance the cytoplasmic and periplasmic concentrations of unwanted transition metals by entering the cellular arena and going into competition for metal cations with the “frivolous” metal uptake systems. Interestingly, all three types of metal efflux systems seemed to possess some degree of frivolity toward metal ions as well, albeit perhaps not as outspoken as for the metal uptake systems. The *C. metallidurans* CzcD exporter (Rmet_5979), for instance, allowed as a CDF protein Zn^2+^, Cd^2+^, or Co^2+^ as a substrate [54], whereas the DmeF and FieF exporters (Rmet_0198 and Rmet_3406) displayed as CDF family members broad metal specificity for Zn^2+^, Cd^2+^, Co^2+^, and Ni^2+^ [55]. It is worth mentioning that disruption of the *dmeF* gene in strain CH34 dramatically lowered the resistance for Co^2+^ (but not for Zn^2+^, Cd^2+^, and Ni^2+^), indicating a complex interplay between the DmeF exporter and the CzcCBA and CnrCBA efflux pumps (possibly partially obscured by the action of other metal resistance systems) [55]. Moreover, CDF proteins can play diverse roles and may possess different metal ion selectivity depending on the environmental conditions (i.e., by adjusted K_d_ values for certain metals) [56]. In addition, the eight metal resistance-related P_1B_-type ATPases currently identified in strain CH34 can be subdivided into two groups according to their substrate profile [28]: Those that extrude Cu^+^ and Ag^+^ (CupA and CupF) and those that extrude Zn^2+^, Cd^2+^, Co^2+^, or Pb^2+^ (ZntA, CadA, PbrA, and CzcP). These exporters mainly differ in the presence of unique amino acid sequences in their transmembrane MBDs, hence defining their metal specificity. But even within a subgroup, differences may exist in terms of metal affinity. For example, CzcP encoded by plasmid pMOL30 is unable to mediate Zn resistance on its own but rather augments the metal exportability of the ZntA, CadA, and PbrA exporters [57]. In a similar fashion, the five active HME-RND efflux systems in strain CH34 displayed a limited substrate spectrum, pumping out either the monovalent metal cations Cu^+^ and Ag^+^ (CusA, SilA) or the divalent metal cations Zn^2+^, Ni^2+^, and Co^2+^, with occasionally also Cd^2+^ (ZniA, CnrA, CzcA) [28] (Figure 2). In such HME-RND systems, two steps of heavy-metal extrusion were discerned, the periplasmic and the transenvelope efflux (Figure 2). Each step involved the interaction of metals with MBDs within the Membrane Fusion Protein (MFP) and RND proteins. Sometimes, the delivery of periplasmic metal ions to the typical C_3_B_6_A_3_-complex is facilitated by a small periplasmic metallochaperone, as is the case for the *E. coli* CusCBFA system [58] (and likely, based on CusF aa sequence similarities, also the CusCBAF complex of strain CH34). Little is known about the substrate specificity of the metal-binding proteins of HME-RND efflux complexes. Apparently, metal-induced conformational changes in the C_3_B_6_A_3_-complex are required in order to create a proper metal-guiding C_3_B_6_A_3_ channel for metal export to take place [48,59,60,61].

As mentioned, it is not inconceivable that the genetic changes in MSR33 instigated cellular conditions or pleiotropic effects that were generally favourable for Cd^2+^ detoxification and hence led to the observed improvement in Cd^2+^ resistance. Possibly, this involved the temporal recruitment of one or more metal binding export proteins, from known metal resistant systems or from hitherto unknown export systems, able to bind Cd^2+^. The transition metals cadmium and mercury belong to Group 12 of chemical elements in the periodic table, together with zinc and copernicium. Although these four metals differ in significant respects, they also have common properties. Particularly, Cd and Hg are similar in their outer shell electron configuration (d^10^s^2^) and atomic radius (ca. 150 pm), and their cations both have a high affinity for sulfhydryl groups in cellular compounds and proteins (i.e., in methionine and cysteine residues). From this perspective, competition between Cd^2+^ and Hg^2+^ for certain MBDs cannot be excluded. Bacterial evolution and adaptation to new or rapidly changing environments implies a delicate balance between the safeguarding of genome integrity and the tolerance for genome instability. A too-rigid genome inevitably will lead to the demise of innovative power and hence adaptability of the host, whereas a too plastic or “fluid” genome may lead to disadvantageous mutations and cell growth arrest, or even cell death. This balance between beneficiary and perilous change in a bacterial genome also relates to the general fitness and the energy household of its host. Members of the genus *Cupriavidus*, and in particular *C. metallidurans*, appear to be masters in adaptation as they are home to a wide variety of habitats, often in extreme conditions [62,63,64,65]. The introduction of the 54 kb plasmid pTP6 into a strain already carrying two large replicons of 3.9 and 2.6 Mb (chromosome and chromid, respectively) and two megasized plasmids of 171 and 234 kb (pMOL28 and pMOL30, respectively) could be seen as a serious additional burden to the host regardless of whether or not phenotypical or physiological changes occur.

Because the charting of genomic changes in MSR33 with respect to its parental strain CH34 did not provide us with any clues or direct evidence on the involvement of certain genetic loci or of any of the known metal resistance determinants on the observed augmented Cd^2+^ resistance, we decided to compare the basal gene expression data for strains CH34 and MSR33 using RNA microarray technology in an attempt to associate their gene expression profiles with strain-specific physiological behaviour, with a focus on differentially expressed (DE) genes that might be involved in the cellular detoxification of heavy metals such as Cd^2+^.

### 3.3. Transcriptional Analysis of Strain MSR33

Strains CH34 and MSR33, which were equally grown in nonselective conditions without any metal-related stress, were investigated for basal gene expression levels, and their expression profiles were compared. A total of 107 DE genes showed statistically significant changes in their expression (Figure 2). Affected genes pertained to the main chromosome (55 genes), the chromid (36 genes), and the plasmids pMOL30 (3 genes) and pMOL28 (13 genes) (Figure 2). In general terms, the products of these 107 genes could be grouped according to their predicted annotated function [12]: Catalytic function (35 genes), transport (24 genes), transcriptional regulation (11 genes), recombination (6 genes), movement and chemotaxis (9 genes), and miscellaneous (22 genes) (Table S7). Most of these genes had a higher expression in strain MSR33, with only 16 genes in strain MSR33 showing a lower expression level. What is immediately striking is that all genes of the *cnrYXHCBAT* operon on plasmid pMOL28 had a significantly higher expression (Figure 2), with log_2_ fold changes ranging from four to six. As we know from MSR33 whole genome sequence analysis, this high expression of the *cnr* locus in strain MSR33 was the direct result of IS*1088*-mediated *cnrY* inactivation and hence derepression of the *cnr* locus, explaining the increased resistance we observed for strain MSR33 to Co^2+^ and Ni^2+^ (see previous sections). Equally noticeable is the complete absence of an altered expression of the *czc* locus on pMOL30, strongly suggesting that the augmented Cd^+2^ resistance we see for strain MSR33 was independent of this locus. This, in fact, corroborates earlier findings about the pMOL30-less CH34 derivative AE126 (which, like MSR33, also has an IS*1088*-mediated inactivated *cnrY* gene on the remaining plasmid pMOL28): Vandecraen et al. [50] showed, next to a heightened resistance to Zn^2+^, Co^2+^, and Ni^2+^, a 2-fold increased resistance to Cd^2+^. Adding another level of complexity, when strains AE126 and AE104 (a CH34 derivative lacking both pMOL28 and pMOL30 plasmids) [35] were transformed with pTP6, these strains, like MSR33, gained an improvement in Cd^2+^ resistance, albeit to a lesser extent (Rojas LA, personal communication).

This would indicate that the augmentation of Cd^2+^ resistance in MSR33 by pTP6 conjugation should be seen as a layered process brought about by multiple factors and possibly diverse mechanisms supporting each other. We cannot say at this point what these mechanisms precisely are and how and when they are triggered, as we have no information about the genomic changes in pTP6 conjugants of AE104 and AE126 (as pTP6 conjugation in CH34 causes genomic changes, this would most likely also be the case for pTP6 conjugation in strains AE104 and AE126, but not necessarily involving the same genomic changes). Clearly, further studies are needed to understand the augmented metal resistance in pTP6 conjugants of CH34 and its derivatives, including (1) the resequencing of pTP6-conjugated AE104 and AE126 strains and (2) the extensive RNAseq-based genetic response analyses for a wider range of heavy metals in all three pTP6-conjugated strains. The additional possibility that some of the observed genetic changes were already introduced to the recipient CH34 strain prior to conjugation with pTP6 cannot be entirely excluded. Lastly, the plasmid-curing procedures used to obtain strains AE104 and AE126 (i.e., applying mitomycin C, nalidixic acid, or hydroxyurea to growing CH34 cells [35]) may also have had mutagenic effects or may have induced transposition activity. In this respect, it would be best, in the frame of future studies, to resequence these strains as well.

A very high log_2_ fold difference in the expression of >4 was also noted in strain MSR33 for the Rmet_4229 gene, a *dctA* paralogue whose product was functionally annotated as a C4-dicarboxylate transporter and which is unlikely to have any connection to metal detoxification or resistance, and gene Rmet_2382, originally identified in CH34 as a transposase-encoding *tnpA* gene (IS*1088*) (Figure 2). Intermediate high log_2_ fold changes of >2 were seen in strain MSR33 for another 17 genes (Table S7), whereas the remaining 72 genes showed a log_2_ fold change between one and two. None of these genes is thought to be involved in metal binding, metal detoxification, or metal resistance. Among the genes with lowered expression in strain MSR33, we noted the *pelG* gene (Rmet_4161), which is part of the *pelABCDEFG* operon required to produce an extracellular polysaccharide that has been implicated in biofilm development [66]. Our sequence data confirmed that the IS*1088* element transposed into the *pelF* gene (Table 2), thereby disrupting expression of the *pelG* gene. This could explain the complete lack of biofilm formation in strain MSR33 reported to us by P. Alviz in a personal communication.

In conclusion, the genome of MSR33 underwent eight insertions, three deletions, and nine SNPs. At least seven of the insertions were due to the action of mobile genetic elements, with their presence fully confirmed by sequence data (implicating IS*1088* in six cases), whereas one small insertion and all three deletions in strain MSR33 may have been the result of DNA recombination or transposition events. The *C. metallidurans* genome is known to be ridden with a very high number of mobile genetic elements, with 57 IS elements, 19 other transposable elements, and 16 genomic islands for its type strain CH34 [12,32,34]. In concordance with this genomic fluidity, *C. metallidurans* displays a highly versatile metabolism and an inherent ability to inhabit a variety of harsh environments [9,62,63,64,65]. This adaptability has not come about overnight but is the wonderful result of microbial evolution over long periods of time. In a time in which large chunks of DNA were retrieved from the environment (e.g., by plasmid transfer or gene exchanges), adaptation was brought about by DNA mutations and natural selection and molecular inventions took place, steadily moulding the genome into its present large (6.9 Mb) and highly malleable form, providing the bacterium with a vast array of possibilities for rapid genetic responses (hence its well-chosen epithet as “Master Survivalist”) [12]. However, tinkering with this hugely evolved and dynamic genome holds intrinsic dangers. Although the plasmid pTP6 was maintained stably in strain CH34 (i.e., MSR33) for over 70 generations under nonselective conditions [31], it has now become clear from our study at hand that the receiving host’s genome underwent multiple changes in the form of 11 INDELs and 9 SNPs, affecting the physiology and heavy metal resistance of the host. It would be wrong to point the finger at the extra plasmid as the “usual suspect” for these genetic changes, but rather we hold the actual process of conjugation responsible. Conjugative interaction appears to be a strong stimulus for transposition [67,68,69], and hence it is easy to envisage that, as a result of conjugation procedures, some elements of the extensive mobilome of *C. metallidurans* (with nearly 100 mobile elements) were triggered into action and “moved around”, causing genetic changes that led to clearly perceptible but also less visible (and less understood) effects alike. The take-home message here is that the genetic engineering of bacteria with large complex and dynamic genomes should be carried out with much caution and that a strong preference should be given to the new generation of small broad-host-range cloning vectors and CRISPR-based technologies nowadays available [70,71,72,73,74].

## Figures and Tables

**Table 1:** Minimal inhibitory concentration (MIC) of heavy metals for *Cupriavidus metallidurans* strains MSR33 and CH34.

Strain/Metal in mM	Hg^2+^	Cd^2+^	Ni^2+^	Co^2+^
*C. metallidurans* CH34	0.01	2.0	10.0	11.0
*C. metallidurans* MSR33	0.10	4.0	12.0	20.0
*C. metallidurans* CH34 (pBBR::*merTPAGB*_1_)	0.10	2.0	ND ^1^	ND ^1^

^1^ ND: not determined.

**Table 2:** Insertions (INs) and deletions (DELs) present in the genome of *C. metallidurans* MSR33.

Type	Replicon (#)	Start	End	Size (bp)	Description	Targeted Gene(s)	Function (MaGe Annotation) (*)
IN	CHR1	328395	328395	1102	IS*1088*	Rmet_0312	putative transporter
IN	CHR1	3106728	3106728	1102	IS*1088*	Rmet_2857 (*tauB*)	taurine ABC transporter ATP-binding protein
DEL	CHR2	602035	602490	455		Rmet_4033	LysR family transcriptional regulator
IN	CHR2	741818	741818	1102	IS*1088*	Rmet_4160 (*pelF*)	EPS biosynthesis, biofilm formation
IN	CHR2	1529231	1529231	1102	IS*1088*	Rmet_4867 (*acrA*)	membrane fusion protein, multidrug efflux
IN	CHR2	2113815	2113815	256	*tniA*/Tn*3*	Rmet_5388 (*apbE*)	ApbE-like lipoprotein
IN	CHR2	2253560	2253560	3	+CTT	Rmet_5508	long-chain-fatty-acid-CoA ligase
DEL	CHR2	2253566	2253568	3	−CGG	Rmet_5508	long-chain-fatty-acid-CoA ligase
IN	CHR2	2440975	2440975	1104	IS*1088*	Rmet_5682 (*nimB*)	membrane fusion protein, heavy metal transport
IN	pMOL28	53484	53484	1104	IS*1088*	Rmet_6205 (*cnrY*)	antisigma factor
DEL	pTP6	26155	27385	1230		*upf30.5*, *upf31.0*, *parA*	respectively an outer membrane protein, a DNA methylase, and a plasmid partition protein

# Chromosome: CHR1; chromid: CHR2. * Genomic and Metabolics analysis software (www.genoscope.cns.fr).

**Table 3:** Single nucleotide polymorphisms (SNPs) detected in the genome of *C. metallidurans* MSR33.

Replicon (#)	Position	SNP	SNP Type	Affected Gene	Gene Description *
CHR1	333850	A→G	intergenic (+201/−75)	Rmet_0314 →/→ *ssb1*	putative transporter, major facilitator family/single-stranded DNA-binding protein (helix-destabilizing protein)
CHR1	645608	A→G	A23A (GCA→GCG)	Rmet_0598 →	Ser/Thr protein phosphatase family protein
CHR1	2400725	G→A	intergenic (−77/+116)	*greA* ←/← *carB*	transcription elongation factor/carbamoyl-phosphate synthase large subunit
CHR1	3418147	A→G	V295A (GTC→GCC)	*dppF* ←	dipeptide transporter; ATP-binding component of ABC superfamily
CHR1	3444412	A→G	V17A (GTC→GCC)	NirJ ←	heme *d*_1_ biosynthesis protein
CHR1	3456113	A→G	V76A (GTG→GCG)	acyP ←	acylphosphatase
CHR2	2253543	A→T	V158E (GTG→GAG)	Rmet_5508 ←	long-chain-fatty-acid-CoA ligase
CHR2	2253553	G→T	P155T (CCG→ACG)	Rmet_5508 ←	long-chain-fatty-acid-CoA ligase
CHR2	2529357	T→C	G264G (GGA→GGG)	Rmet_5769 ←	esterase

# Chromosome: *CHR1*; chromid: *CHR2*; arrows show positive → or negative ← gene orientation; * www.genoscope.cns.fr.

## Supplementary Materials

The following are available online at http://www.mdpi.com/2073-4425/9/11/551/s1. Figure S1: Genetic map of plasmid pTP6; Table S1: Primers designed for this report; Table S2: Transcriptional changes observed in MSR33 versus CH34 under basal conditions; Table S3: Homology analysis of *mer* gene products present in plasmid pTP6; Table S4: Effect of various mixtures of Hg^2+^ and Cd^2+^ on *C. metallidurans* strains MSR33 and CH34 growth; Table S5: Plasmid copy number (PCN) for *C. metallidurans* strains MSR33 and CH34, determined by quantitative PCR; Table S6: *mer* gene occurrences on the replicons of strains CH34 and MSR33; Table S7: Expression changes of *C. metallidurans* MSR33 with respect to *C. metallidurans* CH34, both grown under nonselective conditions.

## References

[1] Bakermans C. (2015). Microbial Evolution under Extreme Conditions.

[2] Knoll A.H. (2015). Paleobiological perspectives on early microbial evolution. CSH Perspect. Biol..

[3] Springael D., Top E.M. (2004). Horizontal gene transfer and microbial adaptation to xenobiotics: New types of mobile genetic elements and lessons from ecological studies. Trends Microbiol..

[4] Cao L., Liu H., Zhang H., Huang K., Gu T., Ni H., Hong Q., Li S. (2012). Characterization of a newly isolated highly effective 3,5,6-trichloro-2-pyridinol degrading strain *Cupriavidus pauculus* P2. Curr. Microbiol..

[5] Ilori M.O., Picardal F.W., Aramayo R., Adebusoye S.A., Obayori O.S., Benedik M.J. (2015). Catabolic plasmid specifying polychlorinated biphenyl degradation in *Cupriavidus* sp. strain SK-4: Mobilization and expression in a pseudomonad. J. Basic Microb..

[6] Mergeay M., Van Houdt R., Nojiri H., Tsuda M., Fukuda M., Kamagata Y. (2014). Adaptation to xenobiotics and toxic compounds by *Cupriavidus* and *Ralstonia* with special reference to *Cupriavidus metallidurans* CH34 and mobile genetic elements. Biodegradative Bacteria: How Bacteria Degrade, Survive, Adapt, and Evolve.

[7] Nies D.H. (1999). Microbial heavy-metal resistance. Appl. Microbiol. Biotechnol..

[8] Mergeay M., Storz G., Hengge-Aronis R. (2000). Bacteria adapted to industrial biotopes: The metal resistant *Ralstonia*. Bacterial Stress Responses.

[9] Sobecky P.A., Coombs J.M., Gogarten M.B., Gogarten J.P., Olendzenski L.C. (2009). Horizontal gene transfer in metal and radionuclide contaminated soils. Horizontal Gene Transfer: Genomes in Flux.

[10] Pohlmann A., Fricke W.F., Reinecke F., Kusian B., Liesegang H., Cramm R., Eitinger T., Ewering C., Potter M., Schwartz E. (2006). Genome sequence of the bioplastic-producing “knallgas” bacterium *Ralstonia eutropha* H16. Nat. Biotechnol..

[11] Amadou C., Pascal G., Mangenot S., Glew M., Bontemps C., Capela D., Carrere S., Cruveiller S., Dossat C., Lajus A. (2008). Genome sequence of the beta-rhizobium *Cupriavidus taiwanensis* and comparative genomics of rhizobia. Genome Res..

[12] Janssen P.J., Van Houdt R., Moors H., Monsieurs P., Morin N., Michaux A., Benotmane M.A., Leys N., Vallaeys T., Lapidus A. (2010). The complete genome sequence of *Cupriavidus metallidurans* strain CH34, a master survivalist in harsh and anthropogenic environments. PLoS ONE.

[13] Lykidis A., Perez-Pantoja D., Ledger T., Mavromatis K., Anderson I.J., Ivanova N.N., Hooper S.D., Lapidus A., Lucas S., Gonzalez B. (2010). The complete multipartite genome sequence of *Cupriavidus necator* JMP134, a versatile pollutant degrader. PLoS ONE.

[14] Poehlein A., Kusian B., Friedrich B., Daniel R., Bowien B. (2011). Complete genome sequence of the type strain *Cupriavidus necator* N-1. J. Bacteriol..

[15] Cserhati M., Kriszt B., Szoboszlay S., Toth A., Szabo I., Tancsics A., Nagy I., Horvath B., Nagy I., Kukolya J. (2012). De novo genome project of *Cupriavidus basilensis* OR16. J. Bacteriol..

[16] Hong K.W., Thinagaran D.A.L., Gan H.M., Yin W.F., Chan K.G. (2012). Whole-genome squence of *Cupriavidus* sp. strain BIS7, a heavy-metal-resistant bacterium. J. Bacteriol..

[17] Li L.G., Cai L., Zhang T. (2013). Genome of *Cupriavidus* sp. HMR-1, a heavy metal-resistant bacterium. Genome Announc..

[18] Ray J., Waters R.J., Skerker J.M., Kuehl J.V., Price M.N., Huang J., Chakraborty R., Arkin A.P., Deutschbauer A. (2015). Complete genome sequence of *Cupriavidus basilensis* 4G11, isolated from the oak ridge field research center site. Genome Announc..

[19] Wang X.Y., Chen M.L., Xiao J.F., Hao L.R., Crowley D.E., Zhang Z.W., Yu J., Huang N., Huo M.X., Wu J.Y. (2015). Genome sequence analysis of the naphthenic acid degrading and metal resistant bacterium *Cupriavidus gilardii* CR3. PLoS ONE.

[20] Fang L.C., Chen Y.F., Zhou Y.L., Wang D.S., Sun L.N., Tang X.Y., Hua R.M. (2016). Complete genome sequence of a novel chlorpyrifos degrading bacterium, *Cupriavidus nantongensis* X1. J. Biotechnol..

[21] Mergeay M., Monchy S., Vallaeys T., Auquier V., Benotmane A., Bertin P., Taghavi S., Dunn J., van der Lelie D., Wattiez R. (2003). *Ralstonia metallidurans*, a bacterium specifically adapted to toxic metals: Towards a catalogue of metal-responsive genes. FEMS Microbiol. Rev..

[22] Monchy S., Benotmane M.A., Janssen P., Vallaeys T., Taghavi S., van der Lelie D., Mergeay M. (2007). Plasmids pMOL28 and pMOL30 of *Cupriavidus metallidurans* are specialized in the maximal viable response to heavy metals. J. Bacteriol..

[23] Avoscan L., Untereiner G., Degrouard J., Carriere M., Gouget B. (2007). Uranium and selenium resistance in *Cupriavidus metallidurans* CH34. Toxicol. Lett..

[24] Monsieurs P., Moors H., Van Houdt R., Janssen P.J., Janssen A., Coninx I., Mergeay M., Leys N. (2011). Heavy metal resistance in *Cupriavidus metallidurans* CH34 is governed by an intricate transcriptional network. Biometals.

[25] Ben Salem I., Sghaier H., Monsieurs P., Moors H., Van Houdt R., Fattouch S., Saidi M., Landolsi A., Leys N. (2013). Strontium-induced genomic responses of *Cupriavidus metallidurans* and strontium bioprecipitation as strontium carbonate. Ann. Microbiol..

[26] Van Houdt R., Mergeay M., Mergeay M., Van Houdt R. (2015). Genomic context of metal response genes in *Cupriavidus metallidurans* with a focus on strain CH34. Metal Response in Cupriavidus Metallidurans: Volume I: From Habitats to Genes and Proteins.

[27] Vandenbussche G., Mergeay M., Van Houdt R. (2015). Metal response in *Cupriavidus metallidurans*: Insights into the structure-function relationship of proteins. Metal Response in Cupriavidus Metallidurans: Volume II: Insights into the Structure-Function Relationship of Proteins.

[28] Nies D.H. (2016). The biological chemistry of the transition metal “transportome” of *Cupriavidus metallidurans*. Metallomics.

[29] Millacura F.A., Cardenas F., Mendez V., Seeger M., Rojas L.A. (2017). Degradation of benzene by the heavy-metal resistant bacterium *Cupriavidus metallidurans* CH34 reveals its catabolic potential for aromatic compounds. bioRxiv.

[30] Smalla K., Haines A.S., Jones K., Krogerrecklenfort E., Heuer H., Schloter M., Thomas C.M. (2006). Increased abundance of IncP-1 beta plasmids and mercury resistance genes in mercury-polluted river sediments: First discovery of IncP-1 beta plasmids with a complex *mer* transposon as the sole accessory element. Appl. Environ. Microb..

[31] Rojas L.A., Yanez C., Gonzalez M., Lobos S., Smalla K., Seeger M. (2011). Characterization of the metabolically modified heavy metal-resistant *Cupriavidus metallidurans* strain MSR33 generated for mercury bioremediation. PLoS ONE.

[32] Van Houdt R., Monchy S., Leys N., Mergeay M. (2009). New mobile genetic elements in *Cupriavidus metallidurans* CH34, their possible roles and occurrence in other bacteria. Antonie Leeuwenhoek.

[33] Mijnendonckx K., Provoost A., Monsieurs P., Leys N., Mergeay M., Mahillon J., Van Houdt R. (2011). Insertion sequence elements in *Cupriavidus metallidurans* CH34: Distribution and role in adaptation. Plasmid.

[34] Van Houdt R., Monsieurs P., Mijnendonckx K., Provoost A., Janssen A., Mergeay M., Leys N. (2012). Variation in genomic islands contribute to genome plasticity in *Cupriavidus metallidurans*. BMC Genom..

[35] Mergeay M., Nies D., Schlegel H.G., Gerits J., Charles P., Van Gijsegem F. (1985). *Alcaligenes eutrophus* CH34 is a facultative chemolithotroph with plasmid-bound resistance to heavy metals. J. Bacteriol..

[36] Maniatis T., Fritsch E.F., Sambrook J. (1982). Molecular Cloning: A Laboratory Manual.

[37] Kovach M.E., Elzer P.H., Hill D.S., Robertson G.T., Farris M.A., Roop R.M., Peterson K.M. (1995). Four new derivatives of the broad-host-range cloning vector pBBR1MCS, carrying different antibiotic-resistance cassettes. Gene.

[38] Taghavi S., Vanderlelie D., Mergeay M. (1994). Electroporation of *Alcaligenes eutrophus* with (mega) plasmids and genomic DNA fragments. Appl. Environ. Microb..

[39] Lee C., Kim J., Shin S.G., Hwang S. (2006). Absolute and relative qPCR quantification of plasmid copy number in *Escherichia coli*. J. Biotechnol..

[40] Bolger A.M., Lohse M., Usadel B. (2014). Trimmomatic: A flexible trimmer for Illumina sequence data. Bioinformatics.

[41] Li H., Handsaker B., Wysoker A., Fennell T., Ruan J., Homer N., Marth G., Abecasis G., Durbin R., 1000 Genome Project Data Processing Subgroup (2009). The sequence alignment/map format and SAMtools. Bioinformatics.

[42] Quinlan A.R. (2014). BEDTools: The Swiss-army tool for genome feature analysis. Curr. Protoc. Bioinform..

[43] Li H. (2013). Aligning sequence reads, clone sequences and assembly contigs with BWA-MEM. arXiv.

[44] Norberg P., Bergstrom M., Jethava V., Dubhashi D., Hermansson M. (2011). The IncP-1 plasmid backbone adapts to different host bacterial species and evolves through homologous recombination. Nat. Commun..

[45] Siguier P., Gourbeyre E., Chandler M. (2014). Bacterial insertion sequences: Their genomic impact and diversity. FEMS Microbiol. Rev..

[46] Grass G., Grosse C., Nies D.H. (2000). Regulation of the *cnr* cobalt and nickel resistance determinant from *Ralstonia* sp. strain CH34. J. Bacteriol..

[47] Tibazarwa C., Wuertz S., Mergeay M., Wyns L., van Der Lelie D. (2000). Regulation of the *cnr* cobalt and nickel resistance determinant of *Ralstonia eutropha* (*Alcaligenes eutrophus*) CH34. J. Bacteriol..

[48] Kim E.H., Nies D.H., McEvoy M.M., Rensing C. (2011). Switch or funnel: How RND-type transport systems control periplasmic metal homeostasis. J. Bacteriol..

[49] Collard J.M., Provoost A., Taghavi S., Mergeay M. (1993). A new type of *Alcaligenes eutrophus* CH34 zinc resistance generated by mutations affecting regulation of the *cnr* cobalt-nickel resistance system. J. Bacteriol..

[50] Vandecraen J., Monsieurs P., Mergeay M., Leys N., Aertsen A., Van Houdt R. (2016). Zinc-induced transposition of insertion sequence elements contributes to increased adaptability of *Cupriavidus metallidurans*. Front. Microbiol..

[51] Zheng H., Chruszcz M., Lasota P., Lebioda L., Minor W. (2008). Data mining of metal ion environments present in protein structures. J. Inorg. Biochem..

[52] Thilakaraj R., Raghunathan K., Anishetty S., Pennathur G. (2007). In silico identification of putative metal binding motifs. Bioinformatics.

[53] Kirsten A., Herzberg M., Voigt A., Seravalli J., Grass G., Scherer J., Nies D.H. (2011). Contributions of five secondary metal uptake systems to metal homeostasis of *Cupriavidus metallidurans* CH34. J. Bacteriol..

[54] Anton A., Grosse C., Reissmann J., Pribyl T., Nies D.H. (1999). Czcd is a heavy metal ion transporter involved in regulation of heavy metal resistance in *Ralstonia* sp. strain CH34. J. Bacteriol..

[55] Munkelt D., Grass G., Nies D.H. (2004). The chromosomally encoded cation diffusion facilitator proteins DmeF and FieF from *Wautersia metallidurans* CH34 are transporters of broad metal specificity. J. Bacteriol..

[56] Barber-Zucker S., Shaanan B., Zarivach R. (2017). Transition metal binding selectivity in proteins and its correlation with the phylogenomic classification of the cation diffusion facilitator protein family. Sci. Rep..

[57] Scherer J., Nies D.H. (2009). CzcP is a novel efflux system contributing to transition metal resistance in *Cupriavidus metallidurans* CH34. Mol. Microbiol..

[58] Delmar J.A., Su C.-C., Yu E.W. (2014). Bacterial multi-drug efflux transporters. Annu. Rev. Biophys..

[59] De Angelis F., Lee J.K., O’ Connell J.D., Miercke L.J.W., Verschueren K.H., Srinivasan V., Bauvois C., Govaerts C., Robbins R.A., Ruysschaert J.M. (2010). Metal-induced conformational changes in ZneB suggest an active role of membrane fusion proteins in efflux resistance systems. Proc. Natl. Acad. Sci. USA.

[60] Long F., Su C.C., Lei H.T., Bolla J.R., Do S.V., Yu E.W. (2012). Structure and mechanism of the tripartite CusCBA heavy-metal efflux complex. Philos. Trans. R. Soc. Lond. B Biol. Sci..

[61] Pak J.E., Ekende E.N., Kifle E.G., O’Connell J.D., De Angelis F., Tessema M.B., Derfoufi K.M., Robles-Colmenares Y., Robbins R.A., Goormaghtigh E. (2013). Structures of intermediate transport states of ZneA, a Zn(II)/proton antiporter. Proc. Natl. Acad. Sci. USA.

[62] Sota M., Tsuda M., Yano H., Suzuki H., Forney L.J., Top E.M. (2007). Region-specific insertion of transposons in combination with selection for high plasmid transferability and stability accounts for the structural similarity of IncP-1 plasmids. J. Bacteriol..

[63] Leys N., Baatout S., Rosier C., Dams A., s’ Heeren C., Wattiez R., Mergeay M. (2009). The response of *Cupriavidus metallidurans* CH34 to spaceflight in the international space station. Antonie Leeuwenhoek.

[64] Mijnendonckx K., Provoost A., Ott C.M., Venkateswaran K., Mahillon J., Leys N., Van Houdt R. (2013). Characterization of the survival ability of *Cupriavidus metallidurans* and *Ralstonia pickettii* from space-related environments. Microb. Ecol..

[65] Byloos B., Coninx I., Van Hoey O., Cockell C., Nicholson N., Ilyin V., Van Houdt R., Boon N., Leys N. (2017). The impact of space flight on survival and interaction of *Cupriavidus metallidurans* CH34 with basalt, a volcanic moon analog rock. Front. Microbiol..

[66] Vasseur P., Vallet-Gely I., Soscia C., Genin S., Filloux A. (2005). The *pel* genes of the *Pseudomonas aeruginosa* PAK strain are involved at early and late stages of biofilm formation. Microbiology.

[67] Godoy V.G., Fox M.S. (2000). Transposon stability and a role for conjugational transfer in adaptive mutability. Proc. Natl. Acad. Sci. USA.

[68] Christie-Oleza J.A., Lanfranconi M.P., Nogales B., Lalucat J., Bosch R. (2009). Conjugative interaction induces transposition of ISPst9 in *Pseudomonas stutzeri* AN10. J. Bacteriol..

[69] Baharoglu Z., Bikard D., Mazel D. (2010). Conjugative DNA transfer induces the bacterial SOS response and promotes antibiotic resistance development through integron activation. PLoS Genet..

[70] Jain A., Srivastava P. (2013). Broad host range plasmids. FEMS Microbiol. Lett..

[71] Obranic S., Babic F., Maravic-Vlahovicek G. (2013). Improvement of pBBR1MCS plasmids, a very useful series of broad-host-range cloning vectors. Plasmid.

[72] Tian P., Wang J., Shen X., Rey J.F., Yuan Q., Yan Y. (2017). Fundamental CRISPR-cas9 tools and current applications in microbial systems. Synth. Syst. Biotechnol..

[73] Cook T.B., Rand J.M., Nurani W., Courtney D.K., Liu S.A., Pfleger B.F. (2018). Genetic tools for reliable gene expression and recombineering in *Pseudomonas putida*. J. Ind. Microbiol. Biotechnol..

[74] Xiong B., Li Z., Liu L., Zhao D., Zhang X., Bi C. (2018). Genome editing of *Ralstonia eutropha* using an electroporation-based CRISPR-cas9 technique. Biotechnol. Biofuels.

