# Supplementary material for: Unintentional Genomic Changes Endow Cupriavidus metallidurans with an Augmented Heavy-Metal Resistance
Source: Genes (Basel). 2018 Nov 13;9(11):551. doi: 10.3390/genes9110551 (PMC6266692; doi:10.3390/genes9110551)
Supplement: Supplementary file 1 [file genes-09-00551-s001.pdf]

genes

Supplementary Materials

S1 Table. Primers used in this study.

Targeted gene	Replicon	Primer	Sequence (5′- 3′)
merTPAGB1	pTP6	pTP6mer_Fw	CACTATAGGGCGAATTACGGTCTT TTCTGACGTTGG
		pTP6mer_Rv	AAGGGAACAAAAGCTGTTCTAGC CACTTTCGGTTCG
MCS	pBBR1	pBBR1MCS2_GA_Fw	CAGCTTTTGTTCCCTTTAGTGAG
		pBBR1MCS2_GA_Rv	AATTCGCCCTATAGTGAGTCGTAT
cadA	Chromosome	CadA_Fw_CR1	GCACACTCACACATGGCAAG
		CadA_Rv_CR1	CTGAGACACAGGATGGTCCG
zniA	Chromid	ZniA_Fw_CR2	GCGCAGATCACCCAGGAATA
		ZniA_Rv_CR2	ACTATGACCGTTCGACGCTG
ncca	pMOL30	NccA_Fw_P30	AAGAGTGAATGGCCGGATGG
		NccA_Rv_P30	TCTCAATGGGTTGGGTGACG
cnrA	pMOL28	CnrA_Fw_P28	AAATGTCCCGATCACAGTCGG
		CnrA_Rv_P28	GCCCACCACCGTTTCATTG
merG	pTP6	MerG_Fw_PT6	TACAAATGCGGTATGGGCGT
		MerG_Rv_PT6	CGAGGTTGAACTGTGCATCG

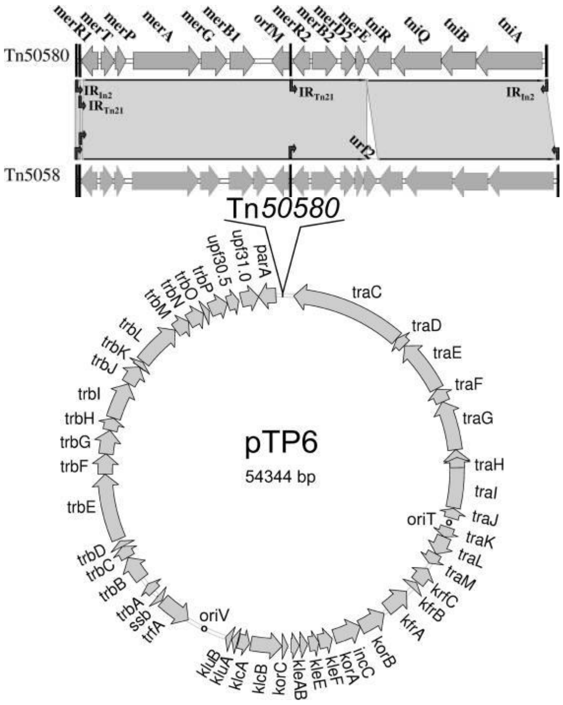

Fig. S1. Genetic map of plasmid pTP6. Figure adapted from Smalla et al., 2006 (Copyright © 2006, Appl Environ Microbiol. 2006 Nov; 72(11): 7253–7259).

S2 Table. Transcriptional changes in MSR33 vs CH34 under equal, non-selective conditions.

	**No genes**	**%Total**
Total genes	6099	100
Pval >0.05	5462	89.56
Pval <0.05	637	10.44
Overexpressed (log_2 >1)	87	0.014
Repressed (log_2 <-1)	15	0.002
Highly overexpressed (log_2 >2)	27	0.004
Highly repressed (log_2 <-2)	1	0.0001

S3 Table. Sequence similarity of *mer* gene products present in plasmid pTP6.

**Gene**	**Protein (aa)**	**Function**	**Organism (Reference)**	**%ID (aa)**
merR1	MerR (144)	activator/repressor of *mer* operon	*C. metallidurans* CH34 (YP_145639.1)	95% (144)
merT	MerT (116)	mercuric ion transport protein	*C. metallidurans* CH34 (YP_145638.1)	87% (116)
merP	MerP (91)	periplasmic mercuric-ion binding protein	*C. metallidurans* CH34 (YP_145637.1)	77% (91)
merA	MerA (569)	mercuric-ion reductase Fad flavoprotein	*C. metallidurans* CH34 (YP_145636.1)	71% (561)
merG*	MerG (217)	organomercurial transporter	*C. testosterone* JL40 (KGH30768.1)	100% (190)
merB-1*	MerB (212)	organomercurial lyase	*B. cepacia* 2a (YP_006965881.1)	100% (212)
merR2	MerR (144)	activator/repressor of mer operon	*C. metallidurans* CH34 (YP_145639.1)	93% (144)
merB-2*	MerB (212)	organomercurial lyase	*B. cepacia* 2a (YP_006965883.1)	100% (212)
merD	MerD (121)	secondary regulatory protein	*C. metallidurans* CH34 (YP_145635.1)	92% (121)
merE	MerE (78)	membrane mercuric resistance protein	*C. metallidurans* CH34 (YP_145634.1)	79% (78)

*Genes not present in *C. metallidurans* CH34

S4 Table: Effect of various mixtures of Hg^{2+} and Cd^{2+} on *C. metallidurans* strains MSR33 and CH34 growth.

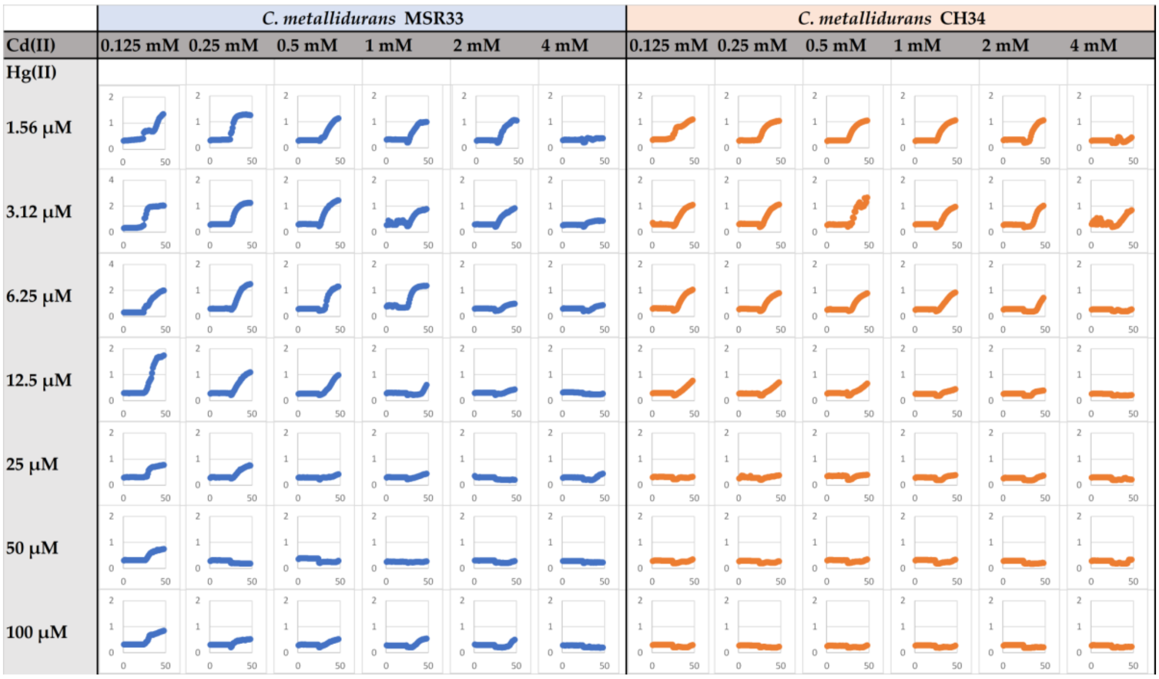

S5 Table. Plasmid copy number (PCN) for *C. metallidurans* strains MSR33 and CH34

	***C. metallidurans* MSR33**				***C. metallidurans* CH34**				
**Replicon (gene)**	**Ct**	**SD**	**Log (DNA)**	**PCN**	**Ct**	**SD**	**log (DNA)**	**PCN**	**Ave-rage PCN**
Chromosome (*cadA*)	23.38	0.05	10.18	1.00	23.47	0.21	10.18	1.00	1.00
Chromid (*zniA*)	20.90	0.19	10.24	1.16	20.18	0.07	10.24	1.14	1.15
pMOL30 (*nccA*)	21.71	0.29	10.26	1.21	19.75	0.20	10.24	1.14	1.18
pMOL28 (*cnrA*)	27.80	0.24	10.25	1.18	24.62	0.07	10.23	1.12	1.15
pTP6 (*merG*)	20.67	0.08	10.50	1.81	35.28	0.60	9.48	0.17	0.99

S6 Table: *mer* gene occurrence on replicons of strains CH34 and MSR33 (individual genes of the *merRT*, *merRTPA*, *merRDE*, *merRTPA* or *merRTPADE* loci are indicated)

Replicon	*mer* genes present in each replicon						PCN*
**CHR1**	*R*	*T*	*P*	*A*			1.00
**pMOL28**	*R*	*T*	*P*	*A*	*D*	*E*	1.15
**pMOL30 – cluster 1**	*R*	*T*	*P*	*A*	*D*	*E*	1.18
**pMOL30 – cluster 2**	*R*	*T*					1.18
**(CH34)**	*4.51*	*4.51*	*3.33*	*3.33*	*2.33*	*2.33*	
**pTP6 – cluster 1**	*R*	*T*	*P*	*A*			1.8
**pTP6 – cluster 2**	*R*				*D*	*E*	1.8
**(MSR33)**	8.11	6.31	5.13	5.13	4.13	4.13	
**Gene unit increase**	+2	+1	+1	+1	+1	+1	
**Gene content increase**	80%	40%	54%	54%	77%	77%	

* PCN values were taken from S5 Table.

S7 Table: Expression changes under non-selective conditions of *C. metallidurans* MSR33 against *C. metallidurans* CH34, represented in a colour coded table.

Movement related genes	Transcriptional regulators	Transporters
Recombinase activity	Catalytic function	Miscellaneous

*Highly over expressed (log_2 ratio > +1) or repressed (log_2 ratio < -1) genes are shown as bold text.

Rmet_code	Gene name	Function	Log ratio
Rmet_0063	*Rmet_0063*	putative allophanate hydrolase subunit 2	-1.67650883
Rmet_0065	*Rmet_0065*	conserved hypothetical protein	-1.23932973
Rmet_0066	*Rmet_0066*	conserved hypothetical protein	-1.37945687
Rmet_0067	*pcp1*	pyrrolidone-carboxylate peptidase	-1.05550107
Rmet_0114	*bioA*	7,8-diaminopelargonic acid synthase, PLP-dependent	-1.42445765
**Rmet_0115**	***bioF***	**8-amino-7-oxononanoate synthase**	**-2.28376218***
Rmet_0116	*bioD*	dethiobiotin synthetase	-1.45848096
Rmet_0117	*bioB*	biotin synthase slightly down RPM proteomics BL (0,72) down also RWV BL (0.68)	-1.4034115
Rmet_0118	*ycdW*	2-ketoacid reductase	-1.55585535
Rmet_0120	*Rmet_0120*	predicted Fe-S protein	-1.23051237
Rmet_0121	*Rmet_0121*	beta-lactamase-like protein	-1.3041744
Rmet_0122	*Rmet_0122*	putative acetyltransferase	-1.23552293
Rmet_0297	*Rmet_0297*	probable nucleoside triphosphate hydrolase domain	1.12141351
Rmet_0310	*Rmet_0310*	putative intracellular protease/amidase/DJ-1/PfpI family	1.11868
Rmet_0410	*rplM*	50S ribosomal protein L13	1.22429

**Rmet_0484**	***tnpB***	**IstB-like ATP binding protein ISRme4**	**2.58535001***
**Rmet_0570**	***modB***	**molybdate transporter subunit ; membrane component of ABC superfamily**	**2.66830376***
Rmet_0599	*Rmet_0599*	cytochrome c oxidase, subunit II	1.91275581
Rmet_0653	*pilA*	Flp/Fap pilin component; Putative pilus subunit protein	2.04791129
Rmet_0775	*phnC*	phosphonate/organophosphate ester transporter subunit; ATP-binding component of ABC superfamily	1.11565916
**Rmet_0777**	***bcr***	**drug resisttransporter Bcr/CflA subfamily**	**2.00829538***
Rmet_0865	*Rmet_0865*	conserved hypothetical protein	1.03606223
Rmet_0887	*Rmet_0887*	putative membrane protein	1.13689678
Rmet_0942	*Rmet_0942*	putative ADP-ribose pyrophosphatase	1.45577081
**Rmet_0986**	***Rmet_0986***	**putative ATPase, AAA family**	**2.01441667***
Rmet_1220	*boxA*	benzoyl-CoA oxygenase component A	1.3183696
**Rmet_1690**	***flhD1***	**DNA-binding transcriptional dual regulator with FlhC**	**2.94450715***
**Rmet_1767**	***Rmet_1767***	**transcriptional regulator, LysR family**	**2.0439383***
**Rmet_1841**	***Rmet_1841***	**4-hydroxybenzoyl-CoA thioesterase**	**2.1924016***
**Rmet_1960**	***phaY***	**D-(-)-3-hydroxybutyrate oligomer hydrolase**	**2.19656841***
Rmet_1980	*ansB*	periplasmic L-asparaginase II	1.74419116
Rmet_2072	*Rmet_2072*	major facilitator superfamily MFS_1	1.06419117
Rmet_2171	*Rmet_2171*	conserved hypothetical protein	1.35381313
**Rmet_2172**	***Int***	**Tyr recombinase activity site-specific recombination Tyr recombinase activity**	**2.82265137***
Rmet_2177	*ppx*	exopolyphosphatase	1.10508189
Rmet_2182	*pstB*	phosphate transporter subunit; ATP-binding component of ABC superfamily	1.00939883
Rmet_2183	*pstA*	phosphate transporter subunit; membrane component of ABC superfamily	1.63382487
Rmet_2185	*pstS*	phosphate ABC transporter periplasmic-binding protein	1.01072
**Rmet_2382**	***tnpA***	**transposase IS1088**	**5.17170573***
**Rmet_2382**	***tnpA***	**transposase IS1088**	**4.72423726***
Rmet_2535	*bug*	extra-cytoplasmic Solute Receptor	1.31844478
Rmet_2737	*Rmet_2737*	hypothetical protein	1.03944721
Rmet_2932	*Rmet_2932*	lactoylglutathione lyase-like protein	1.27848603
Rmet_2995	*ptxA*	phosphonate/organophosphate ester transporter subunit; ATP-binding component of ABC superfamily	1.50871938
**Rmet_3036**	***Rmet_3036***	**conserved hypothetical protein**	**2.0934961***
**Rmet_3126**	***ddlB***	**D-alanine:D-alanine ligase**	**2.27184325***
Rmet_3313	*rpsS*	30S ribosomal subunit protein S19	1.58336143
Rmet_3358	*Rmet_3358*	conserved hypothetical protein	1.54868879
Rmet_3359	*Rmet_3359*	thiamine biosynthesis protein	1.43945338
Rmet_3361	*Rmet_3361*	hypothetical protein; membrane protein	1.11203697

Rmet_3378	*Rmet_3378*	GCN5-related N-acetyltransferase	1.42687412
Rmet_3533	*phhA*	phenylalanine-4-hydroxylase (PAH) (Phe-4-monooxygenase)	1.17501249
Rmet_3534	*phhB*	pterin-4-alpha-carbinolamine dehydratase (transcriptional co-activator)	1.42952206
Rmet_3549	*tctC*	periplasmic tricarboxylate binding receptor (TctC)	-1.2197541
Rmet_3550	*tctB*	component of transporter, tripartite tricarboxylate transport (TTT) family	-1.22615821
Rmet_3589	*acrB*	multidrug efflux system protein	1.1724
Rmet_3628	*Rmet_3628*	hypothetical protein	1.20980184
Rmet_3681	*cheW*	purine-binding chemotaxis protein	1.04754265
**Rmet_3693**	***cheB1***	**chemotaxis response regulator protein-glutamate methylesterase 1**	**2.3968041***
Rmet_3731	*flgN*	flagella synthesis protein FlgN	1.09027262
Rmet_3732	*flgM*	negative regulator of flagellin synthesis (anti-sigma-28 factor, FlgM)	1.2232021
Rmet_3742	*flgJ*	peptidoglycan hydrolase (muramidase)	1.15496048
Rmet_3744	*flgL*	flagellar hook-filament junction protein	1.07665334
Rmet_3826	*Rmet_3826*	alkaline phosphatase	1.46719238
**Rmet_4115**	***Rmet_4115***	**conserved hypothetical protein**	**2.29657966***
Rmet_4161	*pelG*	conserved hypothetical protein	-1.05049624
**Rmet_4299**	***dctA***	**C4-dicarboxylate transport protein 2**	**4.21730516***
Rmet_4332	*tauC*	ABC transporter, permease protein; possible sulfonate/taurine transporter inner membrane component	1.25074713
Rmet_4403	*Shc*	squalene cyclase	1.24741055
Rmet_4404	*shcR*	transcriptional regulator, TetR family	1.21796966
Rmet_4623	*Rmet_4623*	hypothetical protein	1.10951785
Rmet_4664	*Rmet_4664*	phosphoryl transfer system, HPr	1.09003277
Rmet_4726	*Rmet_4726*	diguanylate cyclase/phosphodiesterase	1.52401544
Rmet_4727	*Rmet_4727*	methyl-accepting chemotaxis sensory transducer	1.29962115
Rmet_4834	*ompP*	putative outer membrane pore protein (gram-negative type)	1.27816628
Rmet_4839	*Rmet_4839*	putative transcriptional regulator, LacI family	1.57630345
Rmet_4993	*Rmet_4993*	membrane efflux protein (major facilitator superfamily MFS_1)	1.4303943
Rmet_5075	*musE1*	putative NAD(P)H-dependent FMN reductase SsuE	-1.0976
Rmet_5235	*Rmet_5235*	conserved hypothetical protein; TPR domain protein	1.10058015
Rmet_5238	*Rmet_5238*	conserved hypothetical protein	1.02270936
Rmet_5239	*Rmet_5239*	hypothetical protein (putative dioxygenase)	1.03401905
Rmet_5252	*fliC2*	*fliC2* flagellin	1.84863

Rmet_5279	*Rmet_5279*	transcriptional regulator, LysR family	1.24140191
Rmet_5334	*Rmet_5334*	MgtC/SapB family transporter	1.31906819
**Rmet_5426**	***Rmet_5426***	**conserved hypothetical protein**	**2.78212006***
**Rmet_5559**	***Rmet_5559***	**hypothetical protein**	**2.00853447***
Rmet_5560	*Rmet_5560*	hypothetical protein	1.76768503
Rmet_5620	*Rmet_5620*	conserved hypothetical protein	1.04143379
Rmet_5642	*fliD3*	flagellar hook-associated protein 2	1.23738918
Rmet_5655	*Rmet_5655*	conserved hypothetical protein	1.73791832
Rmet_5855	*wcaF*	glycose-acyl transferase	1.04033538
Rmet_5930	*fusA2*	elongation factor G 2 (EF-G 2)	1.07594255
Rmet_5957	*tnpA*	*orf4*, IS*Rme19*	1.54296414
Rmet_6006	*Int*	Tyr recombinase activity site-specific recombination	1.30660657
Rmet_6171	*merR*	MerR from Tn*4380*, regulatory protein involved in Hg(II) resistance	1.23849795
**Rmet_6196**	***chrP***	**permease of the major facilitator superfamily MFS_1**	**2.14766347***
**Rmet_6205**	***cnrY***	**CnrY, antisigma factor, regulatory protein, involved in Co(II), Ni(II) response**	**5.4418141***
**Rmet_6206**	***cnrX***	**CnrX, antisigma factor, regulatory protein, involved in Co(II), Ni(II) response**	**5.03229334***
**Rmet_6207**	***cnrH***	**CnrH, sigma factor, involved in Co(II), Ni(II) response**	**3.78953232**
**Rmet_6208**	***cnrC***	**CnrC, outer membrane protein, three components cation proton antiporter efflux system, involved in Co(II), Ni(II) resistance**	**4.60736056***
**Rmet_6209**	***cnrB***	**CnrB, membrane fusion protein, three components cation proton antiporter efflux system, involved in Co(II), Ni(II) resistance**	**5.9867383***
**Rmet_6210**	***cnrA***	**CnrA, inner membrane efflux pump, three components cation proton antiporter efflux system, involved in Co(II), Ni(II) resistance**	**5.81023103***
**Rmet_6211**	***cnrT***	**CnrT, Cation Diffusion Facilitator, involved in Co(II), Ni(II) resistance**	**5.3174816***
Rmet_6252	*int*	tyrosine-based site-specific recombinase activity	1.26521262
Rmet_6255	*Rmet_6255*	TetR family transcriptional regulator-like protein	1.18017164
Rmet_6262	*orf112*	Alpha/beta hydrolase	1.22794711
Rmet_6268	*Rmet_6268*	DNA/RNA non-specific endonuclease	1.65810803
Rmet_6300	*trbG*	mating pair formation	1.19131289
